# The perioperative time course and clinical significance of the chemokine CXCL16 in patients undergoing cardiac surgery

**DOI:** 10.1111/jcmm.12708

**Published:** 2015-10-23

**Authors:** Daniela Dreymueller, Andreas Goetzenich, Christoph Emontzpohl, Josefin Soppert, Andreas Ludwig, Christian Stoppe

**Affiliations:** ^1^ Institute of Pharmacology and ToxicologyUniversity HospitalRWTH Aachen UniversityAachenGermany; ^2^ Department for Thoracic and Cardiovascular SurgeryUniversity HospitalRWTH Aachen UniversityAachenGermany; ^3^ Institute of Biochemistry and Molecular Cell BiologyUniversity HospitalRWTH Aachen UniversityAachenGermany; ^4^ Department of Intensive Care MedicineUniversity HospitalRWTH Aachen UniversityAachenGermany

**Keywords:** cardiac surgery, ischaemia and reperfusion, inflammation, chemokines, CXCL16

## Abstract

The chemokine CXCL16 and its receptor CXCR6 have been linked to the pathogenesis of acute and chronic cardiovascular disease. However, data on the clinical significance of CXCL16 in patients undergoing cardiac surgery with acute myocardial ischemia/reperfusion (I/R) are still lacking. Therefore, we determined CXCL16 in the serum of cardiac surgery patients and investigated its kinetics and association with the extent of organ dysfunction. 48 patients underwent conventional cardiac surgery with myocardial I/R and the use of cardiopulmonary bypass (CPB) were consecutively enrolled in the present study. We investigated the peri‐ and post‐operative profile of CXCL16. Clinical relevant data were assessed and documented throughout the entire observation period. To identify the influence of myocardial I/R and CPB on CXCL16 release data were compared to those received from patients that underwent off‐pump procedure. Pre‐operative serum CXCL16 levels were comparable to those obtained from healthy volunteers (1174 ± 55.64 pg/ml *versus* 1225 ± 70.94). However, CXCL16 levels significantly increased during surgery (1174 ± 55.64 *versus* 1442 ± 75.42 pg/ml; *P* = 0.0057) and reached maximum levels 6 hrs after termination of surgery (1174 ± 55.64 *versus* 1648 ± 74.71 pg/ml; *P* < 0.001). We revealed a positive correlation between the intraoperative serum levels of CXCL16 and the extent of organ dysfunction (*r* ^2^ = 0.356; *P* = 0.031). Patients with high CXCL16 release showed an increased extent of organ dysfunction compared to patients with low CXCL16 release. Our study shows that CXCL16 is released into the circulation as a result of cardiac surgery and that high post‐operative CXCL16 levels are associated with an increased severity of post‐operative organ dysfunctions.

## Introduction

Cardiac surgery still belongs to the preferred revascularization strategies in patients with complicated or severe stenosis of the coronary arteries and is annually performed in approximately one million patients worldwide. These numbers are likely to further increase, given the changes in lifestyle, ageing of the population and increasing number of pre‐existing co‐morbidities 1. Patients after cardiac surgery frequently experience disconcerting complication rates, regarding both short‐ and long‐term outcome, which may be due to the systemic inflammatory response with significant release of pro‐inflammatory markers and reactive oxygen species, resulting from the restoration of coronary blood flow and use of CPB 2.

The CXC‐chemokine CXCL16, also called scavenger receptor that binds phosphatidylserine and oxidized lipoprotein, is a transmembrane chemokine, which is expressed on macrophages, epithelial cells, endothelial cells and smooth muscle cells at sites of inflammation 3, 4, 5, 6, 7. The classical stimuli that induce CXCL16 expression are interleukin‐1 (IL‐1) and interferon‐γ, which are both elevated in acute cardiac diseases 8, 9. The transmembrane CXCL16 can act as scavenger receptor and mediate lipid uptake but also binds to the chemokine receptor CXCR6 expressed on leukocytes and thereby mediates cell‐to‐cell adhesion. Moreover, transmembrane CXCL16 is constitutively and inducibly released from the plasma membrane by regulated proteolysis through metalloprotease activity leading to the generation of a soluble chemokine that activates directional migration of CXCR6 expressing leukocytes 10, 11.

During the past two decades, the role of CXCL16 and its receptor CXCR6 in the development of myocardial diseases and atherosclerosis has been controversially discussed. Sheikine and Sirsjo demonstrated significantly reduced serum levels of CXCL16 in patients with coronary artery disease 7, and due to its scavenging function for oxidized lipids and phosphatidylserines, CXCL16 was described to be atheroprotective 12, 13. However, CXCL16 was also shown to be associated with sites predisposed to atherosclerotic lesion development and the formation of foam cells 14, 15. By its ability to recruit and activate CXCR6 expressing leukocytes the chemokine holds a clearly pro‐inflammatory function in acute and chronic heart diseases. In this context, CXCR6 knockout mice showed lower infarction sizes and a reduced risk of atherosclerosis due to decreased tissue recruitment of monocytes and lymphocytes 15, 16. Furthermore, the CXCL16‐CXCR6‐axis is needed for the activation of platelets and their firm adhesion to the vascular endothelium 17, further enhancing plaque formation. In recent studies serum CXCL16 was associated with inflammatory and metabolic risk factors in chronic coronary artery disease and acute coronary syndromes 18, also indicated by C‐reactive protein levels and IL‐8 release in patients with stable or unstable angina 8. The severity of atherosclerosis correlated with serum levels of CXCL16 in metabolic syndrome patients 19 and elevated levels were associated with the development of atherosclerotic ischaemic stroke, especially in large artery atherosclerosis 14, 20. Further studies described the diagnostic function of CXCL16 as a predictive marker for mortality in inflammatory cardiomyopathy, and acute coronary syndromes 21, 22 and circulating serum levels were significantly increased in patients with endocarditis 23 and acute ischaemic stroke 24. However, the significance of CXCL16 in the development of acute organ dysfunctions after myocardial I/R remains elusive. Given the increasing evidence, indicating a pivotal role of CXCL16 in the pathogenesis of cardiovascular diseases, we aimed to study the kinetic and clinical significance of this chemokine in patients undergoing cardiac surgery that represents an ideal clinical model with standardized myocardial I/R and subsequent systemic inflammatory response.

## Materials and methods

### Study design and patients

**Figure 1:**
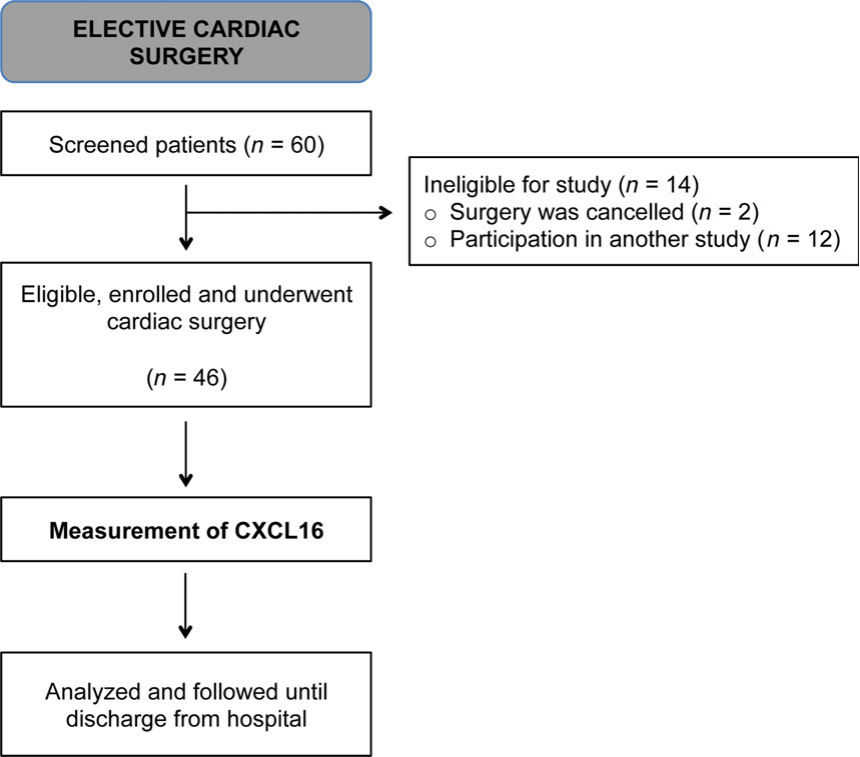
Flowchart according to STROBE‐recommendations for observational studies. From initially screened patients, 46 patients were enrolled in the present study.

46 patients were consecutively enrolled in this prospective observational study (Fig. 1) after approval of the local institutional review board and informed consent. We included all patients scheduled for elective cardiac surgery. Exclusion criteria were known or suspected pregnancy, patients’ age less than 18 years and emergency operations. In addition, serum samples of a cohort of healthy volunteers without any pre‐existing diseases or co‐medications were investigated after written informed consent.

### Management of anaesthesia and surgical procedure

The management of anaesthesia was performed according to our institutional routine and induction of anaesthesia was performed with propofol (1.5 mg/kg) and sufentanil (0.5–1 μg/kg). Muscle relaxation was achieved with rocuronium (1 mg/kg). Maintenance of anaesthesia was executed by continuous infusion of sufentanil (1 μg/kg/hr) and sevoflurane (0.5–1 MAC) during surgery. Basic fluid substitution was performed with 1 ml/kg/hr balanced crystalloid solutions. Packed red blood cells were transfused when the haemoglobin content was below 7.5 g/dl. After termination of surgery, all patients were transferred to intensive care unit (ICU) and the following post‐operative treatment was standardized according to our institutional guidelines.

The surgical procedure with use of conventional CPB (on‐pump) was performed in accordance to our clinical standards. After midline sternotomy, dissection of the internal mammary artery and harvesting of the venous conduits, heparin was administered (300 IE/kg) to obtain an activated clotting time of >400 sec. The extracorporeal circulation was performed with a non‐pulsatile pump flow of 2.2 l/min./m^2^, targeting a blood pressure between 50 and 70 mmHg. A single antegrade infusion of cold crystalloid cardioplegic solution into the aortic root was used for induction of cardiac arrest (CustodiolTM; KöhlerChemie, Alsbach‐Hähnlein, Germany) immediately after cross‐clamping. After weaning from CPB, heparin was antagonized with protamine in a ratio of 1:1, and aspirin was administered p.o. starting 8 hrs post‐operatively.

For performance of off‐pump technique, patients were placed in the Trendelenburg position and rotated to the right to assist in coronary artery exposure and prevent haemodynamic instability during construction of the distal anastomoses. Commercially available mechanical stabilizers were used to facilitate distal anastomoses (Octopus Tissue Stabilizers; Medtronic, Minneapolis, MN, USA). During construction of the anastomoses, an intracoronary shunt was inserted to prevent local myocardial ischaemia and reduce bleeding. Distal anastomoses were performed prior to proximal anastomoses. First anastomosis executed was the left internal thoracic artery to left anterior descending coronary artery anastomosis. Proximal coronary anastomoses were performed during a period of tangential clamping of the ascending aorta. After completion of all anastomoses, heparin was antagonized with protamine in a ratio of 1:1 and aspirin was administered p.o. starting 8 hrs post‐operatively.

### Data collection

Prior to surgery, most relevant demographic and baseline characteristics (*e.g*. age, gender, pre‐existing diseases and co‐medication) were assessed and documented. Further clinical relevant data (concerning surgery and post‐operative outcome) were documented separately. The incidences of organ dysfunction, systemic inflammatory response syndrome, sepsis, severe sepsis and septic shock were assessed and recorded in accordance to the ACCP/SCCM consensus conference criteria 25 and the following organ failure variables were used to determine the incidence of any organ dysfunction 26: arterial hypoxaemia (PaO_2_/FiO_2_ <300 mmHg), coagulation abnormalities in the absence of pharmacological anticoagulation (INR >1.5 or a PTT >60 sec.), hypotension (MAP <70 mmHg) despite adequate fluid replacement, thrombocytopenia (platelet count <100,000/l), hyperbilirubinaemia (plasma total bilirubin >2 mg/dl or 34.2 μmol/l) and neurological dysfunction (Glasgow Coma Score below 15 in absence of sedation or metabolic impairment). Acute kidney injury was defined according to the RIFLE criteria (twofold increase of creatinine or glomerular filtration rate <50% or urine output <0.5 ml/kg/hr for at least 12 hrs).

### Laboratory assessments

Beside the clinical routine measurements, serum samples were collected before induction of anaesthesia, shortly after termination of surgery (admission to ICU) as well as 6 hrs, and 24 hrs after termination of surgery for the determination of CXCL16 concentrations. To further characterize the influence of cardiac surgery (CBP and myocardial I/R) itself on the release of CXCL16, we additionally measured serum levels of CXCL16 in a randomly selected subgroup of patients (*n* = 19) at three further time points to cover the most potent stimulus for the perioperative CXCL16 release (connection to the CBP, myocardial reperfusion, 1 hr after myocardial reperfusion). All blood samples were immediately centrifuged (900 g, 10 min.) and the supernatants transferred into cryotubes. Serum samples were subsequently stored in aliquots at −80°C until final analysis. Furthermore, blood cells were subjected to leukocyte determination by automatic measurement (Sysmex XN 9000; Hamburg, Germany) as part of the clinical routine.

### Measurement of protein and mRNA levels of CXCL16 in the serum and blood cells of patients

Serum levels of CXCL16 were measured using the DuoSet human CXCL16 Elisa Kit (R&D Systems, Wiesbaden, Germany). Samples were stored at −80°C and diluted 20‐fold after careful thawing. The standard ranged from 0 to 5000 pg/ml with a detection limit of 78 pg/ml. Samples were measured in duplicates as previously demonstrated 27. As cell‐free serum samples were not ultracentrifuged, the overall content of CXCL16 in serum was measured, including soluble CXCL16 and potentially on microvesicles remaining transmembrane CXCL16. The mRNA level for CXCL16 in blood cells was quantified by RT‐qPCR analysis and normalized to the mRNA level of GAPDH (glyceraldehyde‐3‐phosphate dehydrogenase). RNA was extracted from frozen ethylenediaminetetraacetic acid blood samples using the NucleoSpin^®^ RNA Blood Kit (Macheray‐Nagel, Dueren, Germany). RNA was reverse transcribed using RevertAid First Strand cDNA Synthesis Kit (Fermentas, St. Leon‐Rot, Germany) according to the manufacturer's protocol. PCR reactions were performed using LightCycler^®^480 SYBR Green I Master Mix (Roche, Mannheim, Germany) according to the manufacturer's protocol. Following primers were used with the specific primer annealing temperature GI254023Xven in brackets: *CXCL16* forward, tgtctatactacacgaggttcca, *CXCL16* reverse, agcatgtccacattctttgag (60°C); *Gapdh* forward, cggggctctccagaacatcatcc, *Gapdh* reverse, ccagccccagcgtcaaaggtg (66°C). All PCR reactions were run on a LightCycler^®^ 480 System (Roche) with the following protocol: 40 cycles of 10 sec. denaturation at 95°C, followed by 10 sec. annealing at the indicated temperature and 15 sec. amplification at 72°C. Standard curves were determined by a serial dilution of a defined cDNA standard. Data were obtained as cycle crossing point (CP) values and calculated as delta CP values using the LightCycler^®^480 software and used for statistic analysis.

### PBMC isolation and CXCR6 surface expression

Human peripheral PBMC from citrated (0.38%) peripheral blood of healthy volunteers were isolated by sedimentation on ficoll hypaque (Amersham, Freiburg, Germany). PBMC were incubated with 8 μg/ml mouse anti‐CXCR6‐phycoerythrin (R&D Systems) or the respective IgG2b isotype control (R&D Systems) in PBS supplemented with 0.2% bovine serum albumin for 1 hr at 4°C and subjected to FACS analysis (LSR II Fortessa; BD Bioscience, Heidelberg, Germany).

### Chemotaxis assay and CXCR6 expression

PBMC were isolated of fresh blood supplemented with 0.4% calcium citrate of healthy volunteers as described before 28. 3 nM recombinant CXCL16 (Peprotech, Hamburg, Germany) or 10% patient serum diluted in RPMI was used as chemotactic stimulus within a 96‐well transwell plate (Corning, New York, NY, USA). CXCL16 neutralization was performed with the antibody AF976 from R&D Systems. Normal goat IgG (R&D Systems) served as isotype control. Random migration was analysed against non‐conditioned medium. After 3 hrs, the transwell‐insert was removed and cells fixated using 1% paraformaldehyde. Nuclei were Hoechst‐stained, and five microscopic images per well were taken. Migrated cells were automatically counted using ImageJ (Rasband, W.S., ImageJ, U. S. National Institutes of Health, Bethesda, Maryland, USA, http://imagej.nih.gov/ij/, 1997‐2014).

### Statistical analysis

All data were statistically analysed using a commercially available software packages (GraphPad Prism 6.0; Graphpad Software Inc., San Diego, CA, USA, and SPSS 22; IBM, Armonk, USA). Data are presented as mean values ± S.E.M. Normally distributed results of single measurements were compared between the groups using the Student's *t*‐test (two groups). Results of repeated measurements were compared using a repeated measurement analysis of variance (one‐way anova). In case of significant results, post‐hoc testing was performed using the Bonferroni adjustment for multiple measurements or the Dunnett adjustment for comparisons to control value. Non‐parametric single measurements were compared using the Mann–Whitney *U*‐test. For correlation studies, linear regression analysis were used. Proportions were compared using the chi‐squared test. In all cases, a level of *P* < 0.05 was considered statistically significant.

## Results

### Enrolled patients and baseline characteristics

From 60 initially screened patients, 46 were enrolled in the present study and followed until discharge from hospital (Fig. 1). Of these, two patients had to be excluded from the study because surgery was cancelled. Baseline characteristics and intra‐operative data are presented in Table 1. All included patients reflect a representative cohort of patients with coronary artery disease and typically pre‐existing disease but without acute myocarditis or myocardial infarction.

**Table 1:** Baseline characteristics and relevant intraoperative data^a^

Demographic data	All patients (*n* = 46)
Age, years	68 ± 9
Gender, male, *n* (%)	35 (76)
Height, cm	172 ± 8
Weight, kg	81 ± 15
euroSCORE	6 ± 3
Prior or pre‐existing disease	
Hypertension, *n* (%)	31 (67)
Chronic pulmonary disease, *n* (%)	6 (13)
Extracardiac arteriopathy, *n* (%)	7 (15)
Diabetes, *n* (%)	10 (22)
Unstable angina, *n* (%)	1 (2)
Recent myocardial infarction (within 90 days), *n* (%)	12 (26)
Chronic kidney disease, *n* (%)	4 (9)
Liver disease, *n* (%)	0 (0)
LVEF >50%, *n* (%)	38 (82)
LVEF 30–50%, *n* (%)	8 (17)
LVEF <30%, *n* (%)	0 (0)
Intra‐operative data	
Duration of surgery, min.	268 ± 69
Duration of CPB, min.	135 ± 44
Ischemia time, min.	89 ± 36

a Data are presented as mean values. Brackets show the lowest and highest values, respectively.

LVEF: left ventricular ejection fraction; CPB: cardiopulmonary bypass.

### Peri‐operative profile of CXCL16 in patients undergoing cardiac surgery

**Figure 2:**
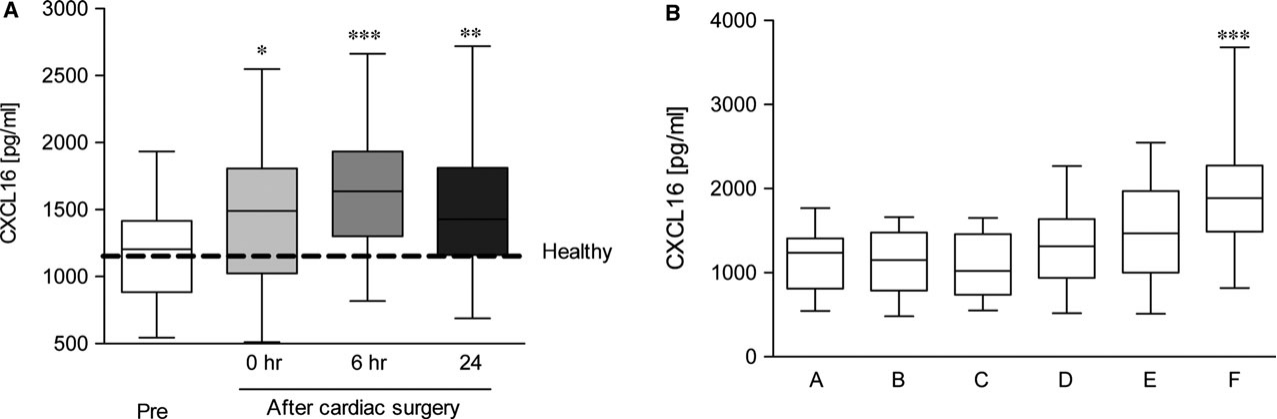
Peri‐operative time course of serum CXCL16 levels. (**A**) Serum samples were taken before surgery (pre) or different time points (0, 6, 24 hrs) after termination of surgery. Shown are mean values ± S.E.M. (**B**) Serum samples of randomly selected patients (*n* = 19) were further analysed for early CXCL16 release during surgery. The following samples were collected: pre‐operative (A), connection to the CBP (B), myocardial reperfusion (C), 1 hr after reperfusion (D), 6 hrs after termination of surgery (E). Shown are mean values ± S.E.M. (A/B) Significance was calculated using one‐way anova and Dunnet post test (*P < 0.05, **P < 0.01, ***P < 0.001; all *versus* pre‐OP).

First, we evaluated the perioperative profile of CXCL16 in all enrolled patients. CXCL16 values and correlations are summarized in Table 2. Baseline values of serum CXCL16 were slightly elevated but still comparable to those obtained from a group of healthy volunteers without any pre‐existing disease (male/female = 50%/50%; age 27 ± 3; *n* = 6). However, serum levels of CXCL16 were significantly elevated directly after termination of surgery and further increased over time, peaking 6 hrs after termination of surgery (Fig. 2A). In this connection, we analysed if this increase may be partly caused by transcriptional regulation and not only by release. Yet, we could not find different mRNA expression in blood cells before surgery and 6 hrs after termination of surgery (Fig. S1). To further characterize the influence of cardiac surgery (CBP and myocardial I/R) itself on the release of CXCL16, samples of 19 randomly selected patients were investigated for the CXCL16 release at several steps of the operation procedure. As shown in Figure 2B, serum levels of CXCL16 did not increase during the operation and were elevated only after the surgical procedure, displaying the overall peri‐operative release of CXCL16. Moreover, CXCL16 release was further elevated 6 hrs after termination of surgery as described above. Thus, the CXCL16 serum level may serve as a rapidly reacting biomarker, which can be easily detected in cardiac surgery patients immediately after surgery.

**Table 2:** CXCL16 values and correlation parameters^a^

CXCL16 values	Mean	SE
Healthy volunteers	1174	55.64
Pre‐operative	1225	70.94
0 h after termination of surgery	1442	55.64
6 h after termination of surgery	1648	74.71
24 h after termination of surgery	1545	98.24
On‐pump pre‐operative	1318	126.4
On‐pump after termination of surgery	2170	268.6
Off‐pump pre‐operative	1297	235
Off‐pump after termination of surgery	2361	356.9

Correlations (CXCL16 *versus* x)	*R* ^2^	*P*‐value
Peri‐operative *versus* organ dysfunction	0.356	0.031
Post‐operative *versus* circulating leukocytes	0.287	0.085
Post‐operative *versus* procalcitonin (PCT)	−0.144	0.711
Post‐operative *versus* CK‐MB	0.148	0.597
Post‐operative *versus* creatinine	0.339	0.033
24 h *versus* blood urea	0.409	0.031

a Data are presented as mean values and S.E. Correlations are described using the correlation coefficient *r* ^2^ and the corresponding *P*‐value.

### Significance of CXCL16 on post‐operative leukocyte recruitment

**Figure 3:**
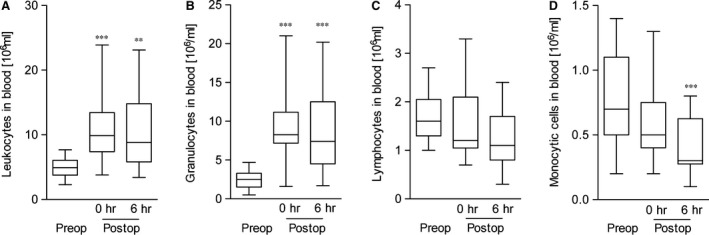
Post‐operative changes of blood cells. (**A**–**D**) The blood leukocyte composition was determined before surgery (preop) as well as directly (0 hr) and 6 hrs after termination of surgery (postop). Shown are mean values ± S.E.M. of leukocytes in **A**, granulocytes in **B**, lymphocytes in **C** and monocytes in **D**. Significance was calculated using one‐way anova and Dunnet post test (**P < 0.01, ***P < 0.001; all *versus* pre‐OP).

**Figure 4:**
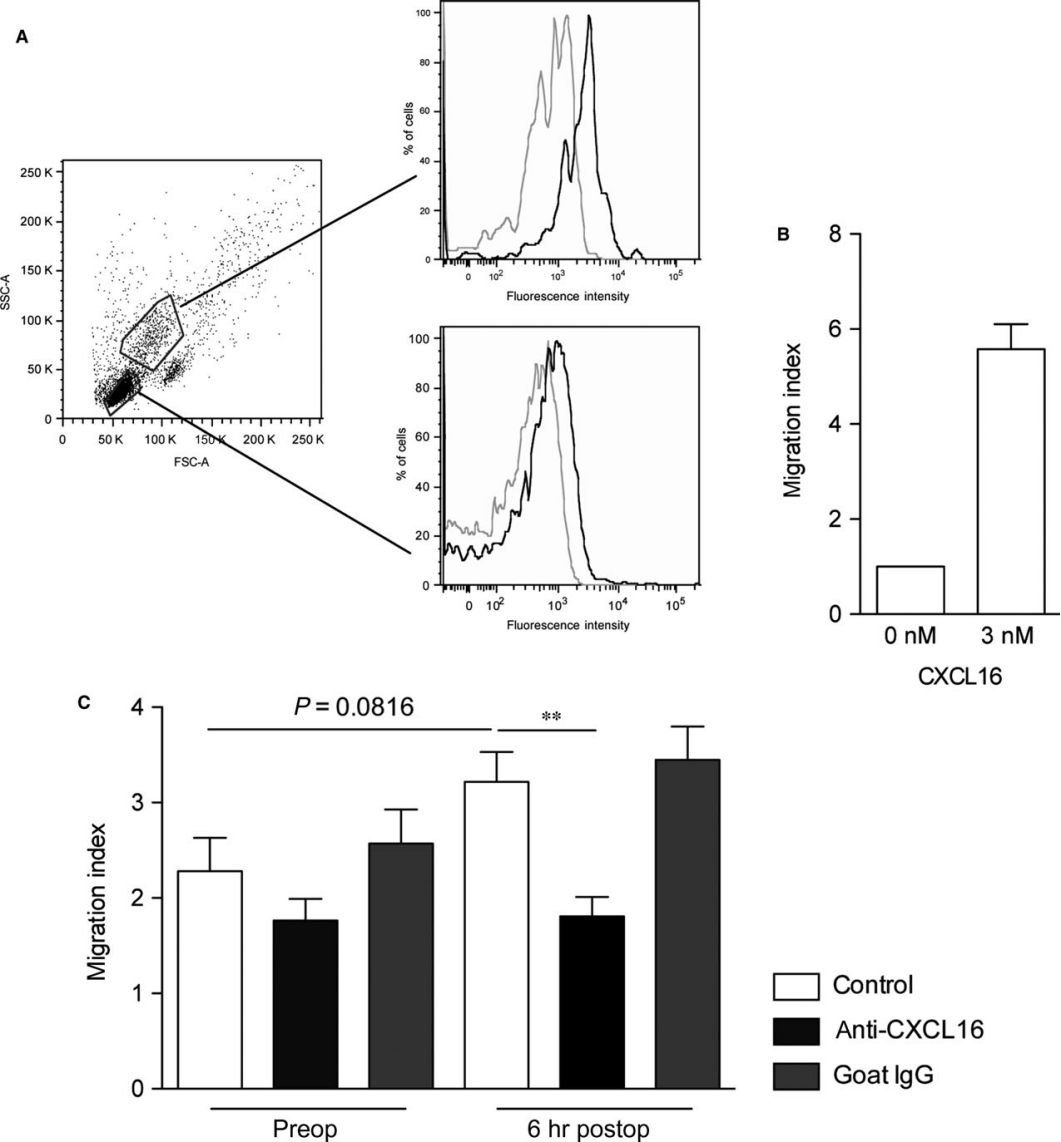
Surgery induced circulating CXCL16 as chemotactic stimulus. (**A**) Surface expression of CXCR6 on lymphocytes and monocytes of freshly isolated PBMCs of healthy volunteers (black line: anti‐CXCR6; grey line: isotype). Shown are representative histograms of three independent PBMC isolations. (**B**) PBMC were subjected to chemotaxis against 3 nM recombinant CXCL16 using a transwell system. (**C**) Comparison of the chemotactic potential of serum from patients before surgery and after termination of surgery (each *n* = 5). Freshly isolated PBMC were used in a transwell system using 10% serum as chemotactic stimulus. The contribution of circulating CXCL16 to the chemotactic potential was shown by a CXCL16‐neutralizing antibody (anti‐CXCL16) in comparison to the isotype control. (**B**/**C**) Shown are mean values ± S.E.M. Significance was calculated using one‐way anova and Dunnet post‐test (**P < 0.01, all *versus* w/o stimulus in **B** and w/o antibody in **C**) and Student's *t*‐test.

Inflammation results in an increase of blood leukocytes and thrombocytes, which may cause tissue damage and systemic inflammatory effects when entering tissue sites 29. CXCL16 is known as a potent chemoattractant and adhesive protein for lymphocytes and monocytes expressing the corresponding receptor CXCR6 11, 29. Therefore, we determined the pre‐ and post‐operative number of blood leukocytes as well as the number of circulating cells 6 hrs after termination of surgery, when serum CXCL16 levels were peaking (compare Fig. 2B). Circulating leukocytes showed a significant perioperative increase (Fig. 3A) that was mainly due to an increased number of granulocytes (Fig. 3B), including neutrophils. In contrast, lymphocytes showed a slight reduction 6 hrs after termination of surgery (Fig. 3C), and the number of monocytes strongly decreased (Fig. 3D). Due to the mentioned chemotactic and adhesive action of CXCL16 and the shown changes in blood cell composition, we assessed the potential associations between CXCL16 levels and the number of thrombocytes and leukocytes after termination of surgery. No correlations were found between the serum levels of CXCL16 and thrombocytes, whereas we noticed a potential association between CXCL16 serum levels and the number of circulating leukocytes after termination of surgery (*r* ^2^ = 0.287; *P* = 0.085). To further determine the chemotactic potential of serum CXCL16, we performed chemotaxis assays with freshly isolated PBMC including lymphocytes and monocytes, which were both shown to express the corresponding receptor on its cell surface (Fig. 4A). PBMC efficiently migrated in response to recombinant CXCL16 (Fig. 4B). Migrated cells were identified as lymphocytes and monocytes by flow cytometry (data not shown). Finally, the migration of these cells towards patient serum samples obtained after termination of surgery was enhanced compared to serum taken before surgery. Importantly, this significant response was reduced by a CXCL16‐neutralizing antibody, whereas the isotype control did not show any effect (Fig. 4C). In this connection, previous studies showed a correlation between pro‐inflammatory cytokines and markers of vascular inflammation and circulating metalloprotease substrates 30. Therefore, we evaluated the potential associations between procalcitonin as well‐established marker of inflammation and CXCL16 in enrolled patients, but no significant correlation was detected (*r* ^2^ = −0.144, *P* = 0.711).

### Clinical relevance of CXCL16 on post‐operative outcome of patients

**Figure 5:**
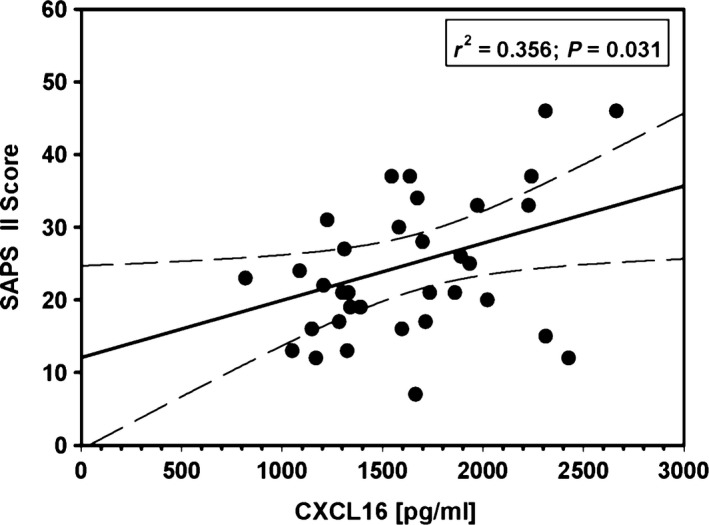
Correlations between circulating CXCL16 levels and SAPS II score on the first POD. Data are depicted as linear regression (black line) with 95% confidence intervals (long dashed line).

The clinical significance of CXCL16 has recently been demonstrated in patients with acute inflammatory cardiomyopathy 21. As the extent of myocardial damage is of high relevance for patients’ outcome (Table 3) and patients’ mid‐ to long‐term perspective, we evaluated the potential association between circulating CXCL16 levels after surgery and CK‐MB, which serves as established marker of myocardial damage. However, no correlations were observed for all enrolled patients (*r* ^2^ = −0.148, *P* = 0.597). To further analyse the relevance of CXCL16 on the post‐operative outcome of patients, we evaluated the clinical significance of circulating CXCL16 levels in patients during cardiac surgery using the SAPS II Score, which is a well‐established organ failure score that adequately represents the extent of organ dysfunction 31. Importantly, we revealed a positive correlation between the post‐operatively measured CXCL16 serum levels and the SAPS score on the 1st post‐operative day (Fig. 5). Considering the significance on single organ dysfunctions, which were evaluated in accordance to the ACCP/SCC criteria 25, we observed a positive correlation between the post‐operative CXCL16 serum levels and post‐operative creatinine levels (*r* ^2^ = 0.339; *P* = 0.033) as well as an association between high levels of CXCL16 and blood urea measured 24 hrs after surgery (CXCL16_24h_ × blood urea: *r* ^2^ = 0.409; *P* = 0.031), indicating that high CXCL16 serum levels could contribute to the development of post‐operative kidney dysfunctions.

**Table 3:** Clinical outcome^a^

Type of organ failure	All patients (*n* = 46)
Atrial fibrillation, *n* (%)	13 (28)
Stroke, *n* (%)	1 (2)
Post‐operative delirium, *n* (%)	13 (28)
Acute kidney injury, *n* (%)	2 (4)
Pneumonia, *n* (%)	3 (7)
Wound infections, *n* (%)	1 (2)
Sepsis, severe sepsis, *n* (%)	0 (0)
SAPS II score	25 (7–71)
Other outcome‐parameters	
Duration of sedation, hrs	19 ± 25
Duration of mechanical ventilation, hrs	17 ± 10
ICU length of stay, hrs	61 ± 66
Hospital length of stay, days	15 ± 10
Overall mortality (*Exitus letalis*), *n* (%)	0 (0)

a Data are presented as mean values. Brackets show percentage min to max.

ICU: intensive care unit.

To further investigate the significance of CXCL16 release on the post‐operative occurrence of organ dysfunctions, we identified those patients with high *versus* low CXCL16 response, as measured by the CXCL16 levels 6 hrs after termination of surgery in relation to the pre‐operative values (mean increase 1.453 ± 0.4481). Patients were separated into two groups with serum levels of CXCL16 higher than the mean values (high responder, CXCL16 increase >mean value) and patients with low CXCL16 levels (low responder, CXCL16 increase <mean value, exemplarily shown in Fig. S2). Although the incidence of major organ complications was reduced in the low responder in comparison to the high responder group, this effect did not reach statistical significance (Table 4). However, we noticed a significantly higher SAPS II score in the ‘high responder’ group (31 ± 15 *versus* 22 ± 8; *P* = 0.017, Table 4), indicating a higher extent of organ injury and a potentially pro‐inflammatory role of serum CXCL16 as disease aggravating factor during cardiac surgery.

**Table 4:** Comparison between CXCL16 high and low responder^a^

Type of organ failure	Low responder (*n* = 19)	High responder (*n* = 27)	*P*‐value
Age, years	66 ± 12 [59–76]	69 ± 8 [64–75]	0.247
Gender, male, *n* (%)	14 (66)	21 (84)	
Height, cm	172 ± 6 [170–178]	172 ± 9 [168–182]	0.895
Weight, kg	82 ± 15 [75–94]	80 ± 15 [66–90]	0.497
Post‐operative delirium, *n* (%)	4 (21)	9 (33)	0.284
Atrial fibrillation, *n* (%)	5 (26)	8 (30)	0.538
Acute kidney injury, *n* (%)	0 (0)	2 (17)	0.296
Pneumonia, *n* (%)	0 (0)	3 (11)	0.193
SAPS II score	22 ± 8 [16–30]	31 ± 15 [21–37]	0.017
Other outcome‐parameters			
Duration of CPB, min.	138 ± 52 [97–170]	133 ± 38 [101–166]	0.698
Duration of mechanical ventilation, hrs	15 ± 5 [11–19]	18 ± 12 [11–25]	0.433
ICU length of stay, hrs	48 ± 37 [22–71]	70 ± 80 [23–70]	0.245
Hospital length of stay, days	14 ± 8 [9–19]	16 ± 11 [10–17]	0.593

a Data are presented as mean values. Parentheses show percentage, brackets indicate range between 25% and 75% quartile.

SAPS: Simplified Acute Physiology Score II; ICU: intensive care unit; CPB: cardiopulmonary bypass.

### Off‐pump cardiac surgery as an alternative surgical approach

**Figure 6:**
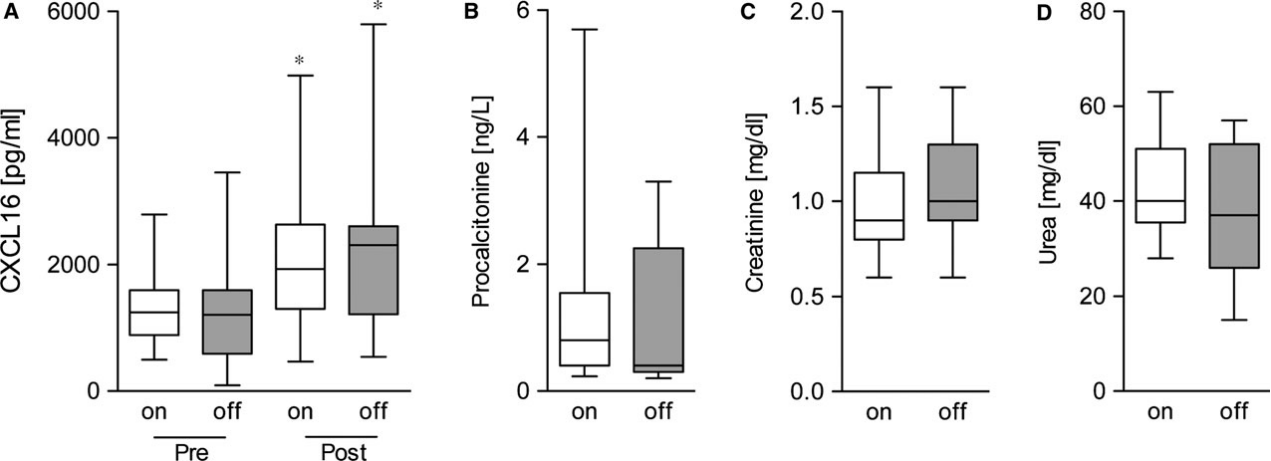
Comparison off‐ *versus* on‐pump‐surgery. Comparison of perioperative inflammation by CXCL16 (**A**) and PCT (**B**) as well as creatinine (**C**) and blood urea (**D**) as markers of renal dysfunction between conventional on‐pump cardiac surgery and off‐pump cardiac surgery with minimized myocardial ischaemia/reperfusion (*n* = 19). Significance was calculated using one‐way anova and Bonferroni post‐test (**A**) or Student's *t*‐test (*P < 0.05).

Cardiac surgery without myocardial I/R and CBP (off‐pump) is considered as a promising approach to reduce the inflammatory response. In this context we investigated if off‐pump surgery reduced the release of the pro‐inflammatory chemokine CXCL16, which should result in a better clinical outcome of patients after cardiac surgery. Therefore, we compared a small cohort of off‐pump surgery patients (66 ± 10 years, 58% male, euroSCORE 5 ± 2) with the aforementioned cohort with respect to the inflammatory response and serum CXCL16 levels. In addition, procalcitonin, creatinine and urea levels were measured as markers of inflammation and kidney injury 24 hrs post‐termination of surgery (Fig. 6). Interestingly, we observed that on‐ and off‐pump‐technique triggered a similar release of CXCL16 after surgery (Fig. 5A), We also observed similar levels of the well‐established inflammation marker procalcitonin (Fig. 6B) as well as postoperative serum levels of creatinine (Fig. 6C) and urea (Fig. 6D). These findings indicate that both techniques provoke a comparable inflammatory response and CXCL16 release, which may result predominantly from the surgical stress.

## Discussion

Despite substantial improvements in the surgical technique and myocardial preservation strategies over the last decades, cardiac surgery still remains associated with disconcerting complication rates that result from imbalance of peri‐operative inflammation. The present study is the first to our knowledge that describes the peri‐operative release profile of the chemokine CXCL16 during cardiac surgery, which is a known key player within the inflammatory response and recently was demonstrated to be elevated in patients with acute myocardial endocarditis 23. The evaluation of the CXCL16 serum content during cardiac surgery revealed a significant up‐regulation of CXCL16 peaking at 6 hrs after termination of surgery. We observed a similar induction of serum CXCL16 levels in patients with on‐pump compared to off‐pump cardiac surgery. The finding that CPB has no further influence on the release of CXCL16 indicates that the surgical procedure itself represents the most prominent stimulus for the release of CXCL16. So far, CXCL16 was only reported in correlation with existing heart and metabolic disorders 15, 19, 21, 22, 23. The present data demonstrate for the first time the function of CXCL16 as critical mediator in cardiac surgery patients. Given the known characteristics of CXCL16 and the early peak after surgery renders this cytokine/chemokine a key player and promising biomarker of the post‐operative inflammatory process, which may trigger metabolic complications and organ dysfunctions after surgery.

The proinflammatory function of CXCL16 in myocardial diseases is supposed to be mainly mediated by interaction with the corresponding receptor CXCR6. Vascular cells express transmembrane CXCL16, whereas the receptor is expressed on leukocytes, especially on monocytes 15, 16, both contributing to the arrest of CXCR6 expressing cells in the blood under flow conditions and recruitment of monocytes to the inflamed sites 32, 33. We observed a post‐operative increase of leukocytes as compared to preoperative values, which corresponds to the observed elevated serum CXCL16 levels. While granulocytes were as well elevated post‐operatively as a mark of systemic inflammation, the total monocyte count showed a post‐operative decrease, which further continued until 6 hrs post‐termination of surgery. In an additional *in vitro* experiment, we could further show that the increase of serum CXCL16 leads to increased migration of monocytes towards serum samples taken from patients after termination of surgery. Therefore, the observed drop in monocytes may reflect the tissue infiltration of CXCR6‐expressing monocytes throughout the post‐inflammatory period.

CXCL16 may not only act as direct chemotactic factor, but also lead to activation of monocytes. This activation could direct the activity of monocytes towards surveillance and reparatory function 34, 35. However, in the blood no changes in the activation status and subtypes of monocytes (CD87, CD14, CD16) were observed after termination of surgery (unpublished observation, personal communication Frank Tacke). Still, in the inflamed tissue those monocytes recruited *via* CXCL16 could also become activated by CXCL16 to present a particular phenotype. In general, an important function of tissue infiltrating monocytes/macrophages after myocardial I/R that may also account for CXCL16 responsive monocytic cells consists in the clearance of dead cells and tissues that subsequently regulate further adaptation mechanisms and fibrosis. Therefore, it remains further elusive which particular phenotype is represented by the described CXCL16‐mediated monocyte recruitment and has to be addressed in further studies.

However, serum levels of CXCL16 may not only reflect the acute inflammation caused by the surgery procedure, but also long‐term systemic side effects, which may lead to the development of postoperative organ dysfunction. Serum CXCL16 was associated with inflammatory and metabolic risk factors in chronic coronary artery disease and acute coronary syndromes 18. Furthermore, it was reported that children with nephrotic syndrome showed elevated levels of serum CXCL16 correlating with blood lipids, urine protein and immune reactions 36, and that CXCL16‐deficient mice were protected against angiotensin II‐induced and blood pressure independent renal injury 37, 38. In accordance with this report we observed a positive correlation between the post‐operative CXCL16 levels and the measured urea and creatinine levels as established markers of renal dysfunction. Furthermore, our study revealed a strong association of CXCL16 serum levels after termination of surgery and the extent of post‐operative organ dysfunctions on the 1st postoperative day, as assessed by the well‐established SAPS II Score 31. Importantly, when analysing the clinical significance of low *versus* high CXCL16 responders after cardiac surgery, we detected a significantly higher extent of organ injury in patients with high CXCL16 serum levels and worse clinical outcome after cardiac surgery. Given the small samples size we were however unable to detect a predictive accuracy of circulating CXCL16 levels. Following analysis in larger cohorts are encouraged to further address this question. Nevertheless, elevated serum CXCL16 levels after surgery may be of particular relevance for the aggravation of disease and the development of renal dysfunctions, which further confirms the supposed key role of serum CXCL16 as useful biomarker in the setting of cardiac surgery.

We questioned whether off‐pump surgery would lower the inflammatory reaction measured by CXCL16 release resulting in a better clinical outcome for the patients. In the past, various studies repeatedly demonstrated that use of CPB frequently triggers an inflammatory response with significant release of various pro‐inflammatory markers 2, which may lead to the development of organ dysfunctions. Therefore, cardiac surgery performed on the beating heart without use of CPB was formally supposed to minimize the postoperative inflammation with resulting complications and promoted to improve postoperative outcome 39, 40. First results were obtained from small cohorts, which confirmed the beneficial effect of off‐pump surgery 39. However, recent data from large‐scale multicenter trial revealed that the incidence of post‐operative organ dysfunctions remained comparable or even increased in patients with off‐pump surgery compared to patients with conventional on‐pump surgery 41. In line with these recent reports, we determined a similar up regulation of the inflammatory response markers including procalcitonin as well as the markers of renal dysfunction in patients with or without CBP. In fact, our findings revealed that also serum CXCL16 levels were comparable in both patient groups. Using serum CXCL16 and the markers of kidney injury as well‐established biomarkers, these findings demonstrates a similar perioperative inflammation in both groups, indicating that off‐pump surgery may not significantly reduce the inflammatory response after surgery.

However, we acknowledge that most data are of correlative nature, which does not explain the underlying mechanisms by which CXCL16 provides its disease aggravating properties. Nevertheless, given the above and in the introduction described characteristics of CXCL16, we suppose that its functions as potent adhesion molecule, chemoattractant and mediator of systemic inflammatory response lead to metabolic disorders and subsequently organ dysfunctions in cardiac surgery patients. However, additional experimental studies, using *in vivo* models are needed to further confirm these preliminary clinical findings and to compare the role of CXCL16 to other pro‐inflammatory cytokines.

Besides, we concede that the small group of included patients may limit the overall significance of the present study. Nevertheless, present data may be considered as hypothesis generating and stimulate further trials to address the role of CXCL16 in the development of organ dysfunction after myocardial I/R and cardiac surgery, evaluating the prognostic value of serum CXCL16 as marker for disease severity and clinical outcome in cardiac surgery patients.

## Conclusion

In conclusion, the present study provides the first data about the kinetics and clinical significance of CXCL16 in patients undergoing cardiac surgery. Cardiac surgery triggered an increase of circulating CXCL16, whereas high serum levels of CXCL16 were associated with the development of organ dysfunctions during the postoperative course. Additional studies with larger cohorts are warranted to establish CXCL16 as a marker for the preoperative risk stratification in patients exposed to myocardial I/R.

## Conflicts of interest

All authors state that no competing financial interests exist.

## Supporting information


**Figure S1** CXCL16 mRNA expression.
**Figure S2** Low‐ and high responder.
