# Supplementary material for: The perioperative time course and clinical significance of the chemokine CXCL16 in patients undergoing cardiac surgery
Source: J Cell Mol Med. 2015 Oct 23;20(1):104–15. doi: 10.1111/jcmm.12708 (PMC4717864; doi:10.1111/jcmm.12708)
Supplement: Supplementary file 1 — Figure S1 CXCL16 mRNA expression. Figure S2 Low‐ and high responder. [file JCMM-20-104-s001.docx]

**Suppl Fig. 1 CXCL16 mRNA expression**

The mRNA expression of CXCL16 was determined in blood cells of patients before surgery and after termination of surgery and expressed in relation to GAPDH as reference gene. Shown are mean values ± SEM. No significant differences were observed.

**Suppl Fig. 2 Low- and high responder**

The increase of CXCL16 over time was calculated by the ratio of preoperative, after termination of surgery or 6 h after termination of surgery, respectively, and preoperative. Plotted are three randomly selected patients of the low responder (open symbols) and high responder (filled symbols) group.
